# Correction: Baláž et al. Mechanochemistry as an Alternative Method of Green Synthesis of Silver Nanoparticles with Antibacterial Activity: A Comparative Study. *Nanomaterials* 2021, *11*, 1139

**DOI:** 10.3390/nano15010049

**Published:** 2024-12-31

**Authors:** Matej Baláž, Zdenka Bedlovičová, Nina Daneu, Patrik Siksa, Libor Sokoli, Ľudmila Tkáčiková, Aneta Salayová, Róbert Džunda, Mária Kováčová, Radovan Bureš, Zdenka Lukáčová Bujňáková

**Affiliations:** 1 Department of Mechanochemistry, Institute of Geotechnics, Slovak Academy of Sciences, Watsonova 45, 04001 Košice, Slovakia; kovacovam@saske.sk (M.K.); bujnakova@saske.sk (Z.L.B.); 2 Department of Chemistry, Biochemistry and Biophysics, University of Veterinary Medicine and Pharmacy, Komenského 73, 04181 Košice, Slovakia; zdenka.bedlovicova@uvlf.sk (Z.B.); patrik.siksa@student.uvlf.sk (P.S.); libor.sokoli@uvlf.sk (L.S.); aneta.salayova@uvlf.sk (A.S.); 3 Advanced Materials Department, Jožef Stefan Institute, Jamova cesta 39, 1000 Ljubljana, Slovenia; nina.daneu@ijs.si; 4 Department of Pharmacology and Toxicology, University of Veterinary Medicine and Pharmacy, Komenského 73, 04181 Košice, Slovakia; 5 Department of Microbiology and Immunology, University of Veterinary Medicine and Pharmacy, Komenského 73, 04181 Košice, Slovakia; ludmila.tkacikova@uvlf.sk; 6 Institute of Materials Research, Slovak Academy of Sciences, 04001 Košice, Slovakia; rdzunda@saske.sk (R.D.); rbures@saske.sk (R.B.)

In the original publication [1], there was a mistake in Figure 6 as published. At the proofreading stage, an incomplete Figure 6a was accidentally inserted in the manuscript. The corrected Figure 6 appears below. The authors state that the scientific conclusions are unaffected. This correction was approved by the Academic Editor. The original publication has also been updated.

## Figures and Tables

**Figure 6:**
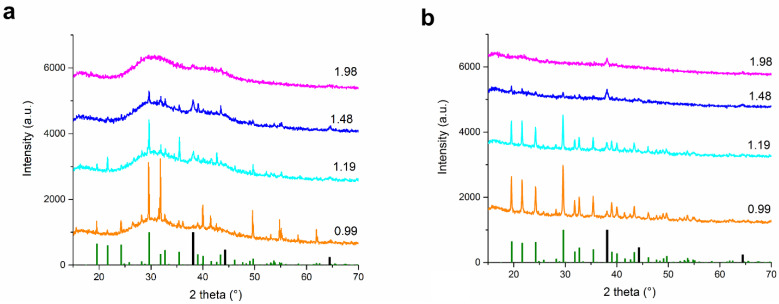
XRD patterns of the dried reaction mixtures after the green synthesis (**a**) and as-milled powders after the mechanochemical synthesis (**b**) using the four highest lavender:AgNO_3_ mass ratio. At the bottom, green bars correspond to orthorhombic AgNO_3_ (ICDD 74-4790) and black to cubic Ag (ICDD 65-2871).
