# Supplementary material for: Mechanochemistry as an Alternative Method of Green Synthesis of Silver Nanoparticles with Antibacterial Activity: A Comparative Study
Source: Nanomaterials (Basel). 2021 Apr 28;11(5):1139. doi: 10.3390/nano11051139 (PMC8146714; doi:10.3390/nano11051139)
Supplement: Supplementary file 1 [file nanomaterials-11-01139-s001.zip › nanomaterials-1175042-supplementary.pdf]

Electronic Supplementary Information for the article

Mechanochemistry as an alternative method to green synthesis of silver nanoparticles with antibacterial activity: A comparative study

Matej Baláž [1*], Zdenka Bedlovičová [2], Nina Daneu [3], Patrik Siksa [2], Libor Sokoli [2], Ľudmila Tkáčiková [4], Aneta Salayová [2], Róbert Džunda [5], Mária Kováčová [1], Radovan Búreš [5], Zdenka Lukáčová Bujňáková [1]

[1] Department of Mechanochemistry, Institute of Geotechnics, Slovak Academy of Sciences, Watsonova 45, 04001 Košice, Slovakia; kovacovam@saske.sk (M.K.); bujnakova@saske.sk (Z.L.B.)
[2] Department of Chemistry, Biochemistry and Biophysics, University of Veterinary Medicine and Pharmacy, Komenského 73, 04181 Košice, Slovakia; zdenka.bedlovicova@uvlf.sk (Z.B.); patrik.siksa@student.uvlf.sk (P.S.); libor.sokoli@uvlf.sk (L.S.); aneta.salayova@uvlf.sk (A.S.)
[3] Advanced Materials Department, Jožef Stefan Institute, Jamova cesta 39, 1000 Ljubljana, Slovenia; nina.daneu@ijs.si
[4] Department of Pharmacology and Toxicology, University of Veterinary Medicine and Pharmacy, Komenského 73, 04181 Košice, Slovakia
[5] Department of Microbiology and Immunology, University of Veterinary Medicine and Pharmacy, Komenského 73, 04181 Košice, Slovakia; ludmila.tkacikova@uvlf.sk
[6] Institute of Materials Research, Slovak Academy of Sciences,04001 Košice, Slovakia; rdzunda@saske.sk (R.D.); rbures@saske.sk (R.B.)
* Correspondence: balazm@saske.sk

UV-Vis Spectra

The time-resolved UV-Vis spectra for the three lavender-richest samples, together with the plot showing the dependence of the absorbance maximum on time for all LEV-Ag-GS samples can be found in Figure S1 a–c and d, respectively.

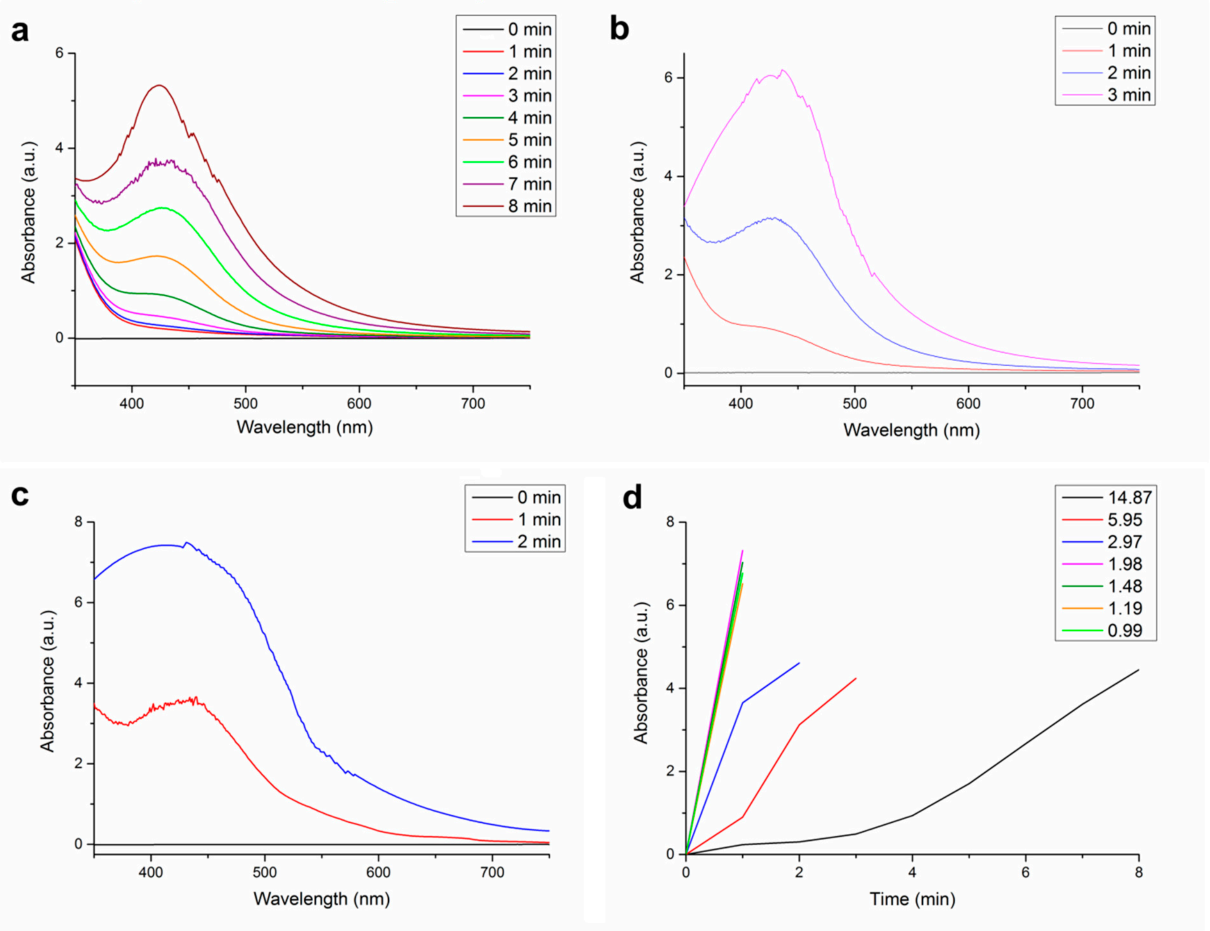

Figure S1. Time-resolved UV-Vis spectra monitoring the green synthesis of Ag nanoparticles using lavender for different lavender : $AgNO_3$ mass ratios: (**a**) 14.87; (**b**) 5.95; (**c**) 2.97; (d) dependence of absorbance on synthesis time for all LEV-Ag-GS samples.

XRD Patterns

The XRD patterns of the as-received powders milled for 15 and 120 min are provided in Figure S2.

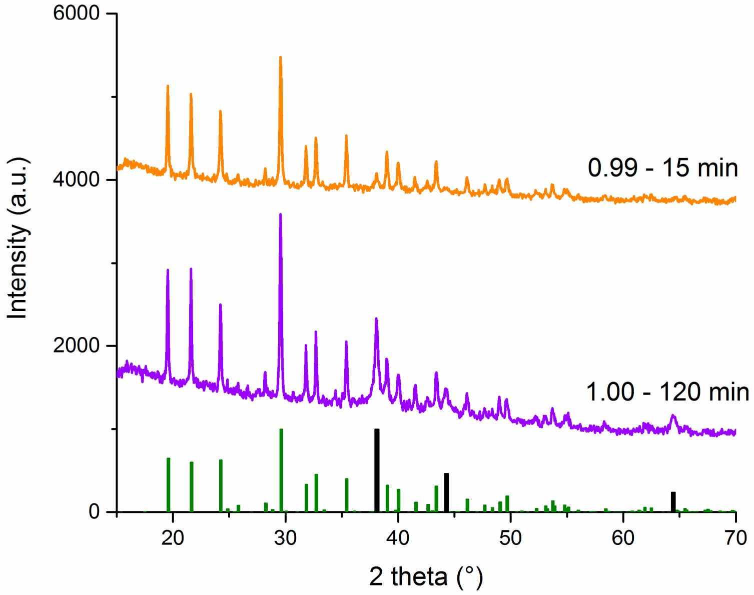

Figure S2. XRD patterns of as-received powders after milling the lavender:$AgNO_3$ ratios 0.99 and 1.00 at 15 and 120 min, respectively. At the bottom, green bars correspond to orthorhombic $AgNO_3$ (ICDD 74-4790) and black to cubic Ag (ICDD 65-2871).

SEM Elemental Mapping

The elemental maps of silver and chlorine in selected regions for LEV-Ag-MSW-5.95 and 0.99 samples are provided in Figure S3.

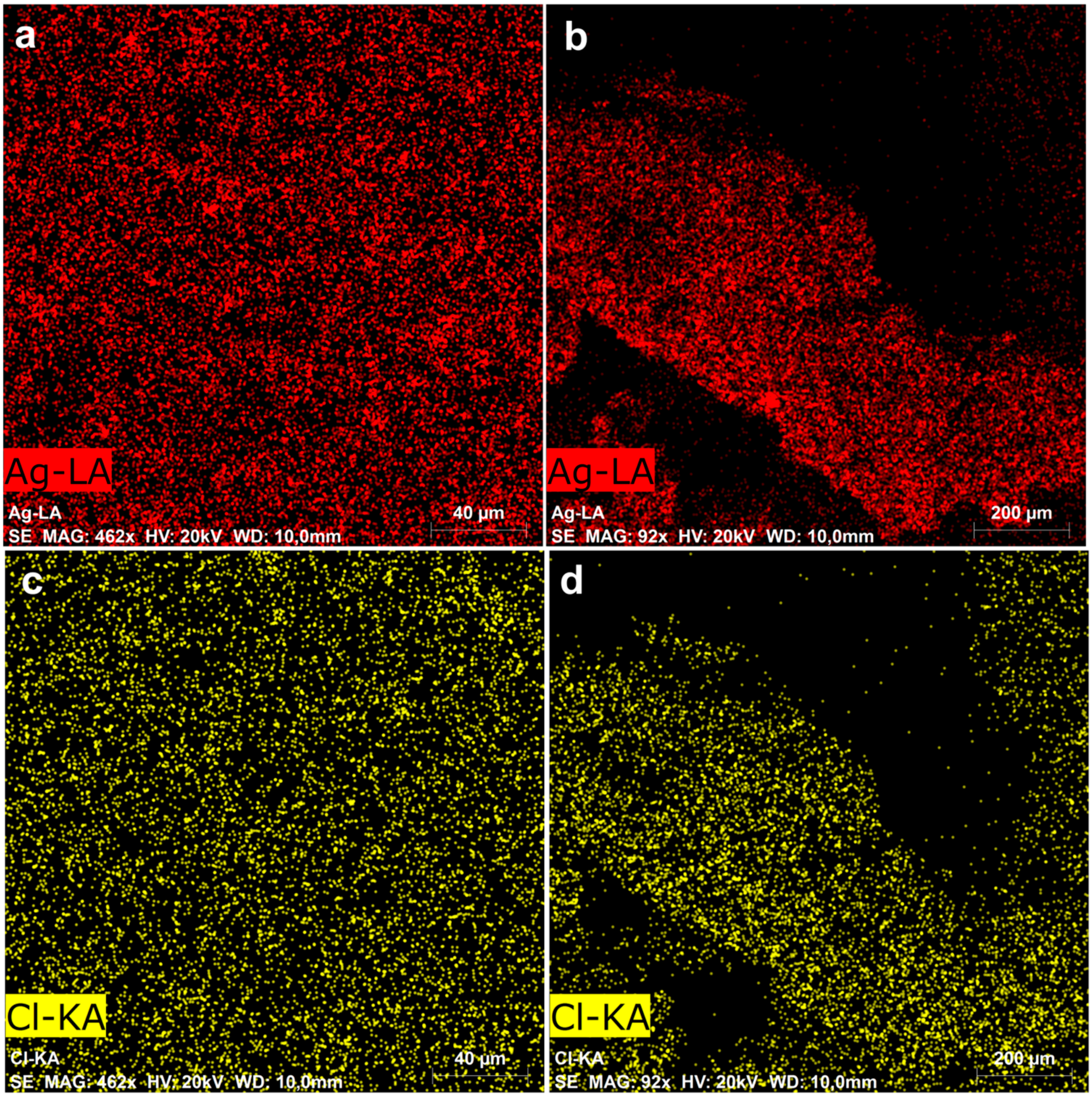

Figure S3. Elemental mapping of LEV-Ag-MSW-5.95 (**a**,c) and 0.99 (**b**,**d**) samples: (**a**, **b**) Ag mapping, (**c**,**d**) Cl mapping.

Grain Size Distribution

To investigate the size of grains obtained using green and mechanochemical synthesis in our case, the photon cross-correlation spectroscopy (PCCS) in wet mode and laser diffraction analysis in dry mode, respectively, were applied. The results are presented in Figure S4.

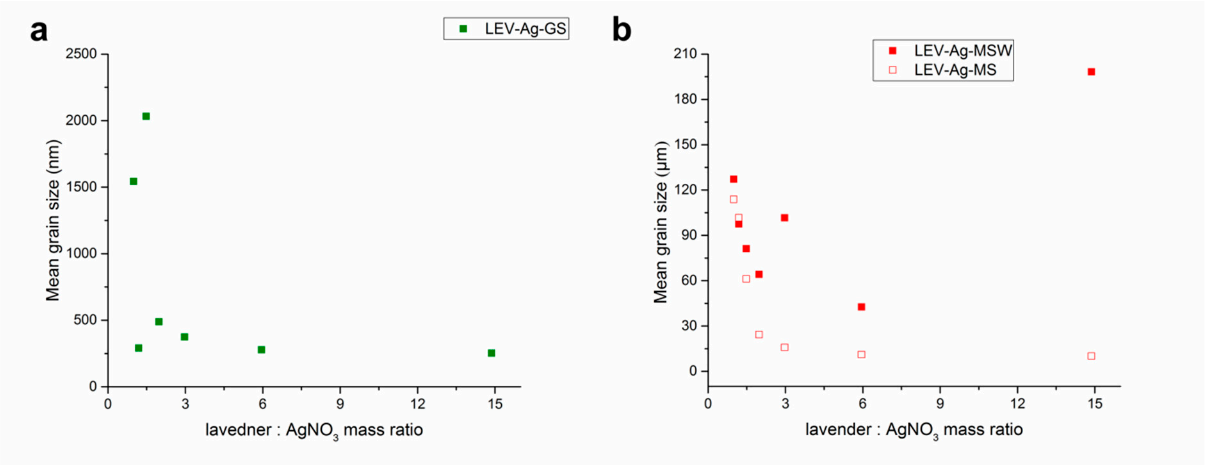

Figure S4. Mean grain size distribution for LEV-Ag products prepared by: (**a**) green synthesis; (**b**) mechanochemical synthesis.

In the case of green synthesis, the PCCS method was used (Figure S4a). In this experimental setup, the grains with large crystallite size immediately sediment at the bottom of the cuvette and many times are not detected by the device. Therefore, the results for low L:A ratios need to be considered with care. For example the x_{50} value of the sample LEV-Ag-GS-1.19 is surprisingly low (291 nm). However, it was possible to observe the huge agglomerates with the naked eye, so this result is not appropriate. The nanosuspensions with higher L:A ratios (starting from 1.98) were homogeneous and no particles could be observed with the naked eye. Thus, the results obtained for these samples should be appropriate. Namely, a constant decrease of the grain size from 488 to 252 nm with increasing L:A ratio from 1.98 to 14.87 has been observed. With regards to uniformity in size, almost unimodal particles were observed only for LEV-Ag-GS-14.87 and 5.95 samples), bimodal distribution was detected for the samples with L:A ratios in the range 2.97–1.19 and the rest of the two samples with lowest L:A ratio exhibited multimodal distribution being a proof of a significant agglomeration.

In the case of mechanochemical synthesis, both as-received powders (MS) and those after washing (MSW) have been analyzed (Figure S4b). Very significant agglomeration into the coarse grains in the case of all samples can be evidenced from the granulometric analysis. In general, the agglomerate size decreases with increasing L:A ratio, in accordance with what has been observed on the nanoscale level for the samples prepared by green synthesis. This is valid for both samples prior and after washing. Thus, the de-agglomerating and stabilizing action of the plant material becomes more significant with the introduction of increased amount of plant. The grain size is always larger after the washing process. The agglomerates produced after milling might have the water-soluble parts of the molecule oriented outside to the environment, whereas the hydrophobic ones might be stabilizing the Ag nanoparticles. When water is introduced, the water-soluble compounds are washed out and the hydrophobic parts join each other to form larger grains. The peculiar result was obtained for LEV-Ag-MS-2.97, namely the grain size of the washed sample was much larger than that of the other ones (with the exception of Ag-LEV-MS-0.99 where the Ag content was very high), so this phenomenon was observed both on nano- (see the crystallite size for Ag^0 reported in Figure 5) and micro-scale. Longer milling for the mixture with the highest amount of $AgNO_3$ (LEV-Ag-1.00-120 min) did not bring about a significant difference with regards to values observed for the samples milled for the short time. However, on the contrary to other samples, larger d_{50} value (146 µm) has been observed prior to washing (the value after washing was 113 µm), so the washing process led to de-agglomeration in this case. Most of the samples exhibited unimodal size distribution with very broad distribution maximum ranging from 10 to 500 µm.

Photographs of Petri Dishes after the Incubation of Bacteria with Ag NPs

To illustrate the positive antibacterial effect, images of the Peteri dishes after the incubation of both LEV-Ag-GS and MDW samples with L:A ratio from 14.87 to 1.19 are provided in Figure S5.

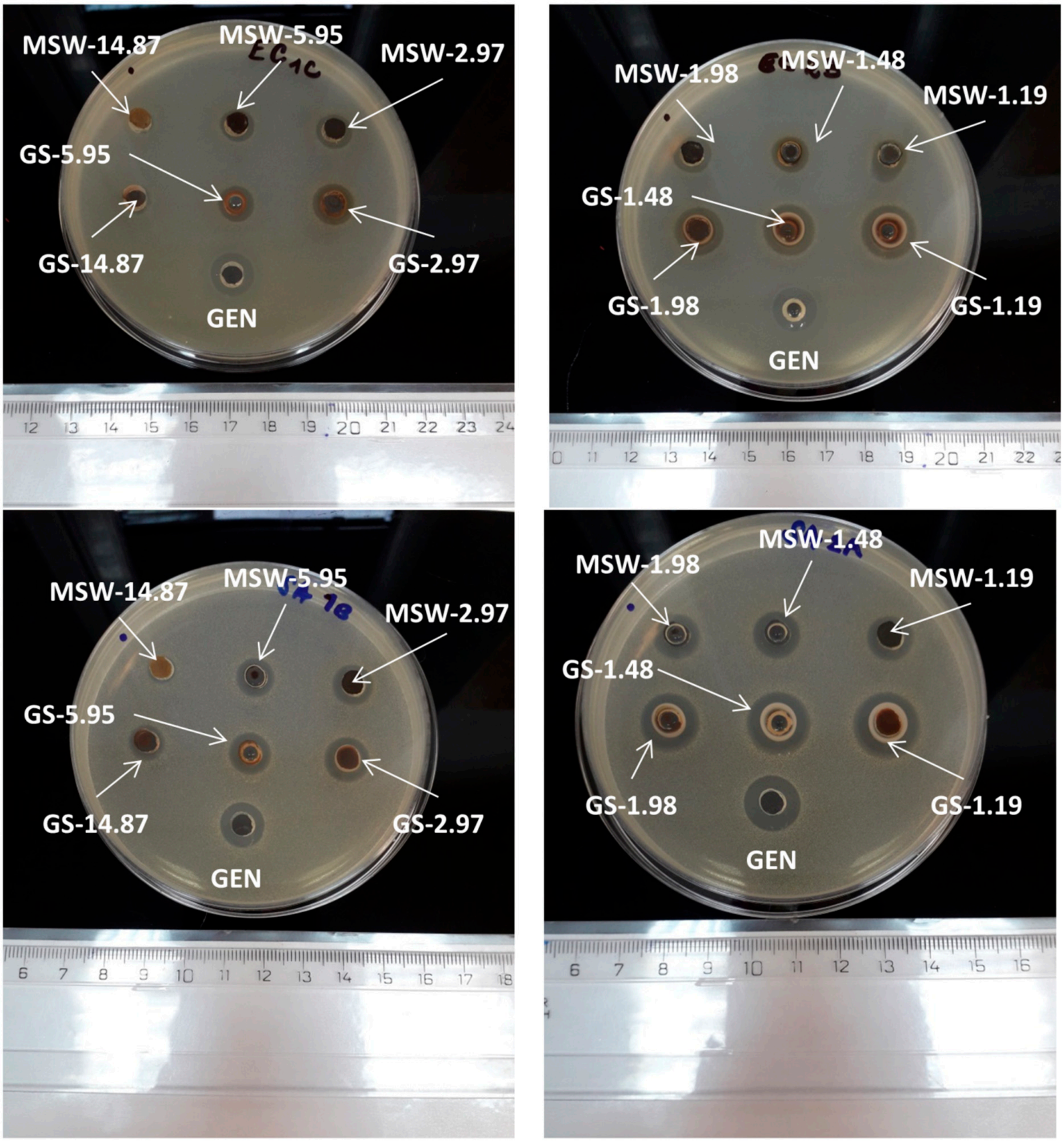

Figure S5. Photographs of Petri dishes after the incubation of bacterial colonies with selected LEV-AG-GS and MSW samples with different L:A ratio. GEN stands for gentamycine.

Antibacterial Activity vs. Amount of Silver in Well

Using quite similar approach to that reported in ref. [43] (cited in the main body of the manuscript), we have calculated the actual amount of Ag present in the well during the antibacterial tests for all samples. For mechanochemically prepared samples, we took the values determined by AAS (Table 2) and calculated the amount of Ag in 20 mg of powder, which was used when preparing the samples for antibacterial tests (it was dispersed in 1 mL distilled water). Then, we have just divided this value by 20, as just 50 μL of suspension were used for test. Similar approach was used for the samples prepared by green synthesis, where the overall volume of reaction mixture was 3 mL. The actual amount of Ag in the reaction mixture is known (it can be easily calculated from the amounts of

$AgNO_3$ stated in Table 1). Again, just 50 μL out of the overall 3 mL were used, so if the amount of Ag is divided by 60, we get its actual content in the well. In Figure S6, these calculated values are plotted against lavender:$AgNO_3$ ratios.

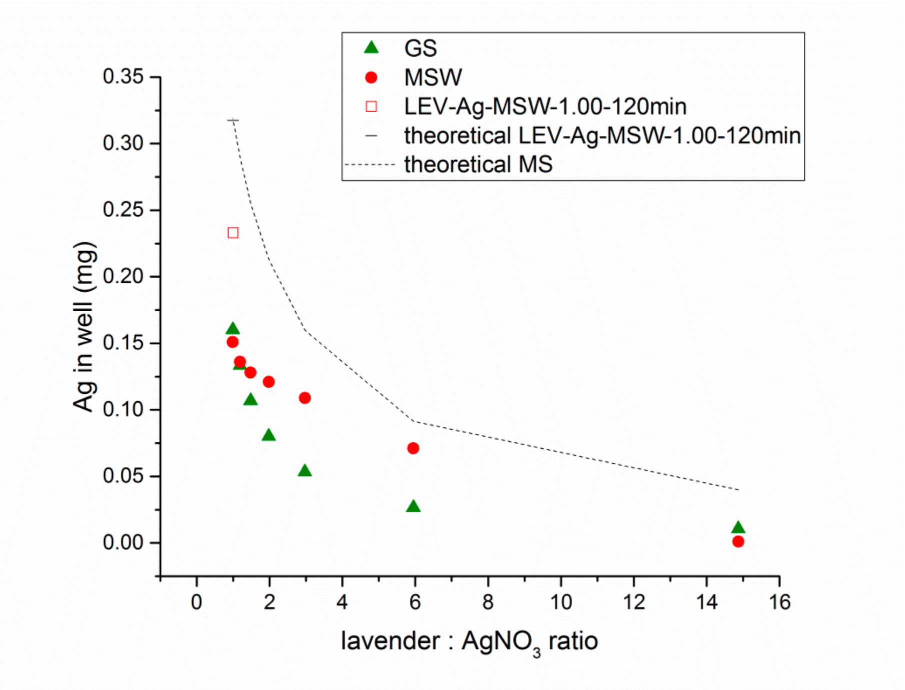

Figure S6. Amount of silver in well in both green and mechanochemically synthesized samples plotted against lavender:$AgNO_3$ mass ratio. Black dashed line corresponds to the theoretical amount of Ag in well, if all silver introduced to the mechanochemical synthesis would be present there.

If we hypothesize that all Ag introduced into the mechanochemical synthesis would be present in the powder subjected to antibacterial tests, the amount of Ag would be higher than in the green synthesis (see comparison of blue points and black dashed line). The difference is getting smaller with the increasing L:A ratio. This is because the amount of lavender was fixed in the green synthesis, whereas it was changed in the mechanochemical synthesis (Table 1). The actual amounts of Ag present in the well for MSW samples were lower than theoretical ones (red points), due to washing out of non-reacted $AgNO_3$ and non-stabilized Ag^0 after the milling process. Upon effective stabilization at higher lavender : $AgNO_3$ ratios, the difference between theoretical and experimental values is getting smaller.

The most important comparison is to compare the antibacterial activities of Ag NPs prepared by MS and GS. Although this has been already shown in Figure 10, it can also be considered taking into account the actual amount of Ag in the well. At low L:A ratios, the actual amount in the well is almost the same for both green and mechanochemically synthesized samples (although its amount can be significantly increased by longer milling). With increasing plant amount and improvement in the stabilization, the amount of Ag in the well becomes higher for the MS samples. For the sample with highest L:A ratio, the situation is inverted, but this is most probably connected with the loss of Ag during the milling process discussed earlier.

In the figure below, the dependence of RIZD values on the actual amount of Ag in the well is shown (Figure S7).

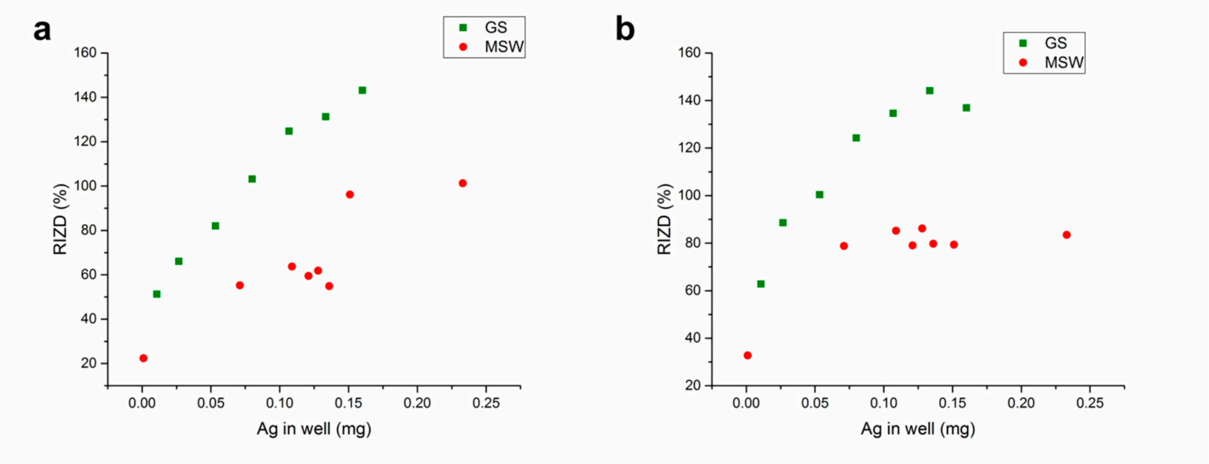

Figure S7. Dependence of relative inhibition zone diameter (RIZD) on the amount of Ag in well: (**a**) *E. coli*, (**b**) *S. aureus*.

It can be clearly seen that the same amount of Ag result in better result in the case of green synthesis. The Ag NPs in the form of nanosuspension prepared by green synthesis are more active due to better diffusion than that in the solid state. The RIZD values nicely correlate with the increase of Ag content in well. However, the contribution of dissolved silver ions coming from non-reacted silver nitrate cannot be neglected, so the final activity is a combination of Ag NPs and Ag^{+} ions. For the MSW samples, the data are much more scattered and furthermore, all these considerations calculate with the fact that the powder has been homogeneously distributed in water prior to being applied into the well. As MSW samples contain micrometric agglomerates (Figure S4b), such phenomenon is quite improbable and the largest agglomerates most probably immediately sediment and are not applied. This is another fact which explains lower antibacterial activity for MSW samples.
